# The Use of Boracic Acid and Calendula in Ear Disease

**Published:** 1882-02

**Authors:** 


### THE USE OF BORACIC ACID AND CALENDULA IN EAR DISEASE.

In an essay read before the Materia Medica Society of New York (*Med. Record*, December 31, 1881) Dr. Samuel Sexton gives an account of the properties of boracic acid and the extract of garden marigold, *calendula officinalis*, when used alone and in combination. The following refers to the employment of these remedies in combination in certain diseases of the ear:

My own observation soon convinced me that the action of calendula on granulating and suppurating tissues, and its prevention of suppuration when used upon recent wounds, would render it a valuable remedy in aural diseases, and I, therefore, subjected the drug to a thorough trial in my practice. The results obtained have been entirely satisfactory. The first trial of it was made with a thin salve, prepared by mixing one drachm of the tincture with three of fluid cosmoline, the alcohol being driven slowly off by the use of a water-bath. This preparation, however, was found not to be satisfactory where dryness of the parts was required, and an attempt was afterward made to saturate absorbent cotton-wool with the tincture, and then evaporate off the alcohol; owing to the gummy nature of the residue retained in the wool, even when a comparatively small quantity of the tincture had been employed, the absorbent quality of the wool was in great measure lost, and this preparation was soon abandoned. It now occurred to the writer that pulverized boracic acid could probably be availed of as a vehicle for carrying the calendula into the ear and retaining it there, and, on trial, the plan proved to to be entirely successful. This mixture is made of equal parts by weight of tincture of calendula and finely powdered boracic acid, as follows: Evaporate the calendula down in a water-bath, at a temperature of about 150° F., to a pasty consistency, and then mix with one-half of the boracic acid; evaporate to dryness, add the other half, and triturate. I do not always employ this preparation in its full strength of the calendula, but frequently find it sufficiently strong when reduced by the addition of more or less pulverized boracic acid.

The local action of both of these remedies on suppurating surfaces seems to be somewhat similar; they are, perhaps, antiseptic, and it is their nature to promote healing; by means of their action secretions are checked, odors are stopped, and healthy granulation promoted. In some otorrhœas the boracic acid may be employed as a dressing, and will not be found so heating as cotton-wool. In this way a light crust is sometimes formed over ulcerating or secreting surfaces, protecting the former while cicatrization goes on, and preventing the latter, when retained, from macerating the neighboring parts and from undergoing decomposition.

In by far the greater number of cases of suppurative otitis media, I prefer these drugs in combination. Their range of application is thus very extensive; when used in traumatic rupture of the drum-head, and in the myringitis accompanying perforative inflammation of the middle ear, the results have been most gratifying. In ulcerative inflammation of the external auditory canal of children, especially in the neglected cases among the poor, where the discharge is sanguineous, purulent, and highly offensive, the mixture is very efficacious.

After the removal of polypi, as soon as the bleeding has been arrested, it is sometimes useful.

The powder may be introduced into the ear through an aural speculum, or it may be blown in with a small insufflator.

In acute cases, the powder should at first be applied with great care, for in some instances it is not well borne at the beginning of the attack.

In nearly all cases, the discharge may be expected to lessen under this treatment; and, when odor is present, its disappearance may be confidently expected to take place very soon.

